# Correction: S1PR1 drives a feedforward signalling loop to regulate BATF3 and the transcriptional programme of Hodgkin lymphoma cells

**DOI:** 10.1038/s41375-019-0511-z

**Published:** 2019-06-25

**Authors:** K Vrzalikova, M Ibrahim, M Vockerodt, T Perry, S Margielewska, L Lupino, E Nagy, E Soilleux, D Liebelt, R Hollows, A Last, G Reynolds, M Abdullah, H Curley, M Care, D Krappmann, R Tooze, J Allegood, S Spiegel, W Wei, C B J Woodman, P G Murray

**Affiliations:** 1 Institute of Cancer and Genomic Sciences, University of Birmingham, Birmingham, UK; 2 Institute of Anatomy and Cell Biology, Georg-August University of Göttingen, Göttingen, Germany; 3 Department of Cellular Pathology, John Radcliffe Hospital, Oxford, UK; 4 Institute of Immunology and Immunotherapy, University of Birmingham, Birmingham, UK; 5 Department of Pathology, Universiti Putra Malaysia, Selangor, Malaysia; 6 Leeds Institute of Cancer and Pathology, University of Leeds, Leeds, UK; 7 Research Unit Cellular Signal Integration, Helmholtz Zentrum München, Neuherberg, Germany; 8 Department of Biochemistry and Molecular Biology and Massey Cancer Center, Virginia Commonwealth University School of Medicine, Richmond, VA USA; 9 Sheffield Institute of Translational Neuroscience, University of Sheffield, Sheffield, UK; 10 Department of Clinical and Molecular Pathology, Institute of Molecular and Translational Medicine, Faculty of Medicine and Dentistry, Palacky University, Olomouc, Czech Republic

**Correction to: Leukemia**

10.1038/leu.2017.275

This article was originally published under a CC BY-NC-ND 4.0 license, but has now been made available under a CC-BY 4.0 license.

